# Body mass index as a predictor of healthy and disease-free life expectancy between ages 50 and 75: a multicohort study

**DOI:** 10.1038/ijo.2017.29

**Published:** 2017-02-21

**Authors:** S Stenholm, J Head, V Aalto, M Kivimäki, I Kawachi, M Zins, M Goldberg, L G Platts, P Zaninotto, L L Magnusson Hanson, H Westerlund, J Vahtera

**Affiliations:** 1 Department of Public Health, University of Turku and Turku University Hospital, Turku, Finland; 2 Department of Epidemiology and Public Health, University College London, London, UK; 3 Finnish Institute of Occupational Health, Turku, Finland; 4 Clinicum, Faculty of Medicine, University of Helsinki, Helsinki, Finland; 5 Department of Social & Behavioral Sciences, Harvard T.H. Chan School of Public Health, Boston, MA, USA; 6 Inserm, Population-based Epidemiologic Cohorts Unit-UMS 011, Villejuif, France; 7 Paris Descartes University, Paris, France; 8 Inserm, Aging and Chronic Diseases. Epidemiological and Public Health Approaches, Villejuif, France; 9 Stress Research Institute, Stockholm University, Stockholm, Sweden

## Abstract

**Background:**

While many studies have shown associations between obesity and increased risk of morbidity and mortality, little comparable information is available on how body mass index (BMI) impacts health expectancy. We examined associations of BMI with healthy and chronic disease-free life expectancy in four European cohort studies.

**Methods:**

Data were drawn from repeated waves of cohort studies in England, Finland, France and Sweden. BMI was categorized into four groups from normal weight (18.5–24.9 kg m^−2^) to obesity class II (⩾35 kg m^−2^). Health expectancy was estimated with two health indicators: sub-optimal self-rated health and having a chronic disease (cardiovascular disease, cancer, respiratory disease and diabetes). Multistate life table models were used to estimate sex-specific healthy life expectancy and chronic disease-free life expectancy from ages 50 to 75 years for each BMI category.

**Results:**

The proportion of life spent in good perceived health between ages 50 and 75 progressively decreased with increasing BMI from 81% in normal weight men and women to 53% in men and women with class II obesity which corresponds to an average 7-year difference in absolute terms. The proportion of life between ages 50 and 75 years without chronic diseases decreased from 62 and 65% in normal weight men and women and to 29 and 36% in men and women with class II obesity, respectively. This corresponds to an average 9 more years without chronic diseases in normal weight men and 7 more years in normal weight women between ages 50 and 75 years compared to class II obese men and women. No consistent differences were observed between cohorts.

**Conclusions:**

Excess BMI is associated with substantially shorter healthy and chronic disease-free life expectancy, suggesting that tackling obesity would increase years lived in good health in populations.

## Introduction

Obesity is a major global public health challenge of our times. The prevalence of overweight and obesity has increased significantly over the past three decades and concerns about the health risks associated with obesity have become nearly universal.^1^ Excess adiposity is associated with an increased risk for morbidity, in particular type 2 diabetes, coronary heart disease, heart failure and several types of cancer^2, 3, 4^ as well as with elevated mortality risk.^5, 6^ In addition, among older people, obesity carries an increased risk of lower extremity osteoarthritis,^7^ and reduced functional capacity and quality of life.^8, 9, 10, 11^ The adverse effects of obesity raise the question about corresponding losses in life spent in good health. This issue has not been examined in detail although it has important implications for both individuals and for societies dealing with increasing health-care costs and care dependency.

A useful summary measure that captures both the 'quantity' and 'quality' of life is the estimation of health expectancy. This concept takes into account both morbidity and mortality and is therefore useful in comparing the health of different populations and population sub-groups.^12, 13^ To date, there are a limited number of studies that have examined how body mass index (BMI) impacts health expectancy.^14, 15, 16, 17, 18, 19, 20, 21^ Moreover, health indicators used to compute health expectancy have varied across studies—for example, single versus multiple chronic diseases,^14, 15, 22^ health-related quality of life,^16^ disability^17, 18, 19^ or quality-adjusted years^20^—which has hindered our ability to draw comparisons across populations.

Some studies^15, 16^ have estimated health expectancy based on cross-sectional methods, that is, prevalence-based life tables of health status, also known as Sullivan's Method.^23^ The advantage of using longitudinal methods, such as multistate life tables (MSLT), in estimating health expectancy is that they take into account the possibility of returning to a state of good health using incident rates instead of prevalence.^24^ When longitudinal data on health development are available, the incidence-based MSLT is preferable because it most accurately reflects the impact of current conditions (that is, disease onset, recovery, and mortality) on the evolution of health expectancy in the target population.^25^

To the best of our knowledge few studies have prospectively studied health expectancy across BMI categories. In these studies healthy years were operationalized in terms of quality-adjusted life years,^20^ years without cardiovascular disease^14, 22^ and disability-free years.^17^ None of the studies used self-rated health which is a global measure of health status and commonly used to estimate health expectancy. In addition, all the above-mentioned studies examined health expectancy for only three broad BMI categories: normal weight, overweight and obesity. With increasing prevalence of severe obesity, more information is needed about health expectancy at the extreme of BMI distribution, that is, obesity and severe obesity, which typically associate with excess morbidity and mortality.^26^

The objective of this study was to address the above-mentioned gaps by examining health expectancy across BMI categories in large cohorts of older men and women living in England, Finland, France and Sweden. In each of the cohorts health expectancy was defined using two different health indicators: sub-optimal self-rated health and having a chronic disease.

## Materials and methods

### Study population

We used data from four prospective cohort studies from England, Finland, France and Sweden to calculate partial life expectancy (LE) and health expectancies between the ages of 50 and 75. In all cohorts, people aged 50 years or older with valid data on health and BMI were included from the first observation. We limited our calculation of partial LE to an upper age of 75 as not all cohorts had participants aged 75 and older and this choice allowed us to have comparable time frames for each cohort.

The English data are from the first six waves (2002–2003 to 2012–2013) of the English Longitudinal Study of Ageing (ELSA), an open-access, nationally representative biennial longitudinal survey of those aged 50 and over living in private households in England who had previously taken part in the Health Survey for England in 1998, 1999 or 2001 (wave 0).^27^ We included 7225 participants aged 50–75 years at baseline who had valid information on objectively measured BMI collected during the home visit at either wave 0 or wave 2.

The Finnish data are from five waves of the Finnish Public Sector study (FPS). The FPS, established in 1997/1998, comprises all 151 901 employees with ⩾6 month job contract in any year from 1991/2000 to 2005 in 10 towns and 5 hospital districts in Finland. Survey data has been collected by repeated surveys at 4-year intervals on all 103 866 cohort members, who were working in the participating organizations during the surveys in the years 1997/1998, 2000/2001, 2004/2005, 2008/2009 and/or 2012/2013 or had retired or left the organizations after the 2000/2001 survey. For the analysis we used data from 42 702 participants aged 50–75 years at the first wave for which valid data on BMI was available.

The French data are from the GAZEL Cohort Study, established in 1989 among Électricité de France-Gaz de France (EDF-GDF) workers, the French national utility company, with annual waves of data collection up to 2014. At inception in 1989, the GAZEL Cohort Study contained 20 625 volunteers (15 011 men and 5614 women) working at EDF-GDF who were then aged from 35 to 50 years.^28^ We included 14 967 participants who had valid information on BMI measured in 1996 and who were aged 50–57 years at the first wave.

Data for Sweden came from five waves of the Swedish Longitudinal Occupational Survey of Health (SLOSH).^29^ The first wave of SLOSH in 2006 was a postal questionnaire follow-up of all respondents to the 2003 Swedish Work Environment Survey (SWES), a cross-sectional, biennial survey of a random stratified sample of those gainfully employed people aged 16–64 years. At wave 2 in 2008, the sample was increased by adding the respondents from the 2005 SWES yielding an overall sample of *n*=18 915 women and men originally representative of the working population in Sweden in 2003 and 2005. These people were then re-surveyed in 2010, 2012 and 2014. In total, 77% responded at least once. The analytic sample in the present study comprised the 8048 participants who had responded to at least one SLOSH wave and who were aged 50–75 years at the first wave for which valid data on BMI was recorded.

In all cohorts, participants gave their informed consent to take part. Ethical approval was given in each of the countries from relevant ethical committees/boards.

### Measurement of BMI

BMI was calculated using self-reported body weight and height in FPS, GAZEL and SLOSH. In ELSA, body weight and height were measured by a study nurse in the participants' homes. Obesity was categorized according to World Health Organization cutoff criteria as (1) underweight (BMI <18.5 kg m^−2^), (2) normal weight (18.5–24.9 kg m^−2^), (3) overweight (25–29.9 kg m^−2^), (4) obese class I (30–34.9 kg m^−2^) and (5) obese class II (⩾35 kg m^−2^).^30^ Because the proportion of underweight men and women in each cohort was less than 1% with the exception of GAZEL women (3%), the underweight people were excluded from the analyses.

### Outcome measures

In each study cohort, we defined two health expectancy outcomes: (1) healthy LE using self-rated health and (2) chronic disease-free LE based on the occurrence of chronic diseases. In addition, we took mortality into account.

#### Self-rated health

All participants were asked about their health status at each wave. Responses were categorized into good and sub-optimal health. In ELSA, FPS and SLOSH participants were asked to rate their general health on a five-point Likert scale, which was dichotomized by categorizing response scores 1–2 as good health and scores 3–5 as sub-optimal health. GAZEL used an 8-point Likert scale (1=very good, 8=very poor), which was dichotomized by categorizing response scores 1–4 as good health and scores 5–8 as sub-optimal health, as previously validated.^31^ Health expectancy based on self-rated health is labeled hereafter as healthy LE.

#### Chronic diseases

Presence of the following chronic diseases was ascertained in each study by asking ‘has a doctor ever told you that you have …':^1^ heart disease (heart attack, coronary heart disease, angina, congestive heart failure, or other heart problems),^2^ stroke (stroke or transient ischemic attack),^3^ chronic lung disease (chronic bronchitis or emphysema or asthma),^4^ cancer (cancer or a malignant tumor of any kind except skin cancer) and^5^ diabetes (diabetes or high blood sugar). Individuals were defined as having a chronic disease if they reported one or more of these conditions. The presence of chronic diseases at baseline (first observation included in analysis) included any chronic diseases reported before the age of 50 from available information on respondents. Health expectancy based on chronic diseases is hereafter labeled as chronic disease-free LE.

Mortality was ascertained from linked register data for each study cohort with follow-up censored on 31 December of the year in which data collection last took place for each study cohort.

### Statistical analyses

Characteristics of the participating cohorts are presented at the first observation point, which refers to the date each participant was included in the dataset.

We applied multistate models to longitudinal data to obtain transition probabilities between health states. Discrete-time multistate life table models were used to estimate partial LE and healthy LE and chronic disease-free LE between the ages of 50 and 75 years (in total 26 years). For both outcome measures, three health states were defined: healthy, unhealthy and dead. For healthy LE, there were four possible transitions between the health states, namely: healthy to sub-optimal health (onset), sub-optimal health to healthy (recovery), healthy to dead, sub-optimal health to dead. For chronic disease-free LE, there were only three possible transitions as, by definition, recovery was not possible.

For each study cohort, age-specific transition probabilities by sex and BMI were estimated from multinomial logistic models with age (in years), sex and socioeconomic position as covariates. Partial LE, healthy LE and chronic disease-free LE from ages 50–75 years were then calculated based on these estimated transition probabilities using a stochastic (microsimulation) approach.^32^ For each study, individual trajectories for a simulated cohort of 100 000 persons were generated with distributions of covariates at the starting point based on the observed study-specific prevalence by five year age group, sex, socioeconomic position and BMI. Partial LE, healthy LE and chronic disease-free LE from age 50 to 75 were then calculated as the average from these trajectories for BMI and sex. Computation of 95% confidence intervals (CI) (from 2.5th and 97.5th percentiles) for these multistate life table estimates was performed using a bootstrap method with 500 replicates for the whole analysis process (multinomial analysis and simulation steps). As BMI-related transitions to poor health and death may differ by sex, we repeated analyses including interactions between sex and BMI in the multinomial logistic models. Finally, since tobacco smoking is associated with increased risk of mortality and morbidity^33, 34^ and it is also typically associated with lower weight,^35^ we conducted sensitivity analyses among never smokers using a bootstrap method with 50 replicates.

All analyses were conducted in SAS 9.2 using the SPACE (Stochastic Population Analysis of Complex Events) program.^36, 37^ This program uses the stochastic (that is, microsimulation) approach to estimate the healthy LE as opposed to another well-known program, IMaCh (Interpolation of Markov Chains) which uses a deterministic approach.^38^

## Results

Characteristics of the study cohorts for men and women at the time of first observation are shown in Table 1. Prevalence of obesity (BMI⩾30 kg m^−2^) varied across cohorts. Obesity was most common among English men (24%) and women (29%) in ELSA, followed by Finnish and Swedish men and women being around 15% in FPS and SLOSH. Obesity was rare among French men and women in GAZEL cohort (7%). At baseline, there were also some differences in the prevalence of sub-optimal self-rated health across study cohorts ranging among men from 19% (GAZEL) to 37% (FPS) and among women from 20% (SLOSH) to 34% (FPS). Chronic disease was most common among ELSA men (34%) and women (31%) and least common in SLOSH men (22%) and women (17%).

Partial LE between ages 50 and years 75 (in total 26 years) for men was 23.7 years in ELSA, 24.1 years in FPS, 24.5 years in GAZEL and 25.2 years in SLOSH. The corresponding figures for women were 24.4 years for ELSA, 24.9 years for FPS, 25.0 years for GAZEL and 25.5 years for SLOSH. On the basis of the most recent national records, the total LE at age 50 for men in England was 31.3, in Finland 30.3, in France 30.8 and in Sweden 32.0 years. Corresponding figures for women were 34.4 for England, 35.0 for Finland, 36.2 for France and 35.1 for Sweden. Thus, the differences in country-level differences in total LE at age 50 were consistent with the cohort-specific partial LEs that we observed.

**Figure 1:**
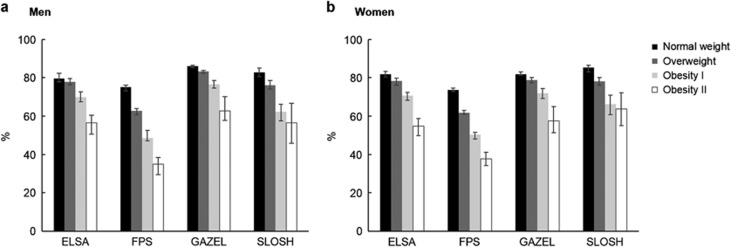
Proportion (95% CI) of life spent in good health between ages 50 and 75 by body mass index in each study cohort. (**a**) Men, (**b**) Women.

Table 2 shows estimates of partial LE between ages 50 and 75 years, divided into healthy and unhealthy LE based on self-reported health and BMI. The partial LE was highest among normal weight and overweight participants and lowest among obese class II participants across the study cohorts. There was a gradient toward shorter healthy LE with increasing BMI. Normal weight men and women could expect to live on average 81% of their life between 50 and 75 years in good health. The corresponding figure was only 64% among obese class I and 53% among obese class II participants (Figure 1). In terms of absolute number of years, normal weight participants from the ELSA, GAZEL and SLOSH cohorts could expect to live on average 3 and 7 years longer in good health compared to those in obesity classes I and II, respectively. In the FPS cohort, the gradient from normal weight to obese class II was slightly larger than in other cohorts, the corresponding differences in healthy years being 6 and 10 years, respectively.

**Figure 2:**
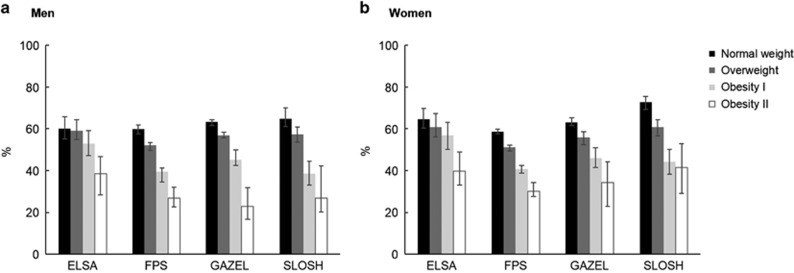
Proportion (95% CI) of life spent without chronic disease between ages 50 and 75 by body mass index in each study cohort. (**a**) Men, (**b**) Women.

Results for the partial LE, chronic disease-free LE and LE with chronic diseases are shown in Table 3. The proportion of years without chronic diseases between ages 50–75 years ranged from 60 (ELSA and FPS) to 65% (SLOSH) in normal weight men and from 59 (FPS) to 73% (SLOSH) in normal weight women (Figure 2). In ELSA the difference between normal weight and obesity classes I and II was 2 and 5 years in men and 2 and 6 years in women, respectively. In all other cohorts, the loss in healthy years among obese classes I and II participants compared to normal weight participants was much larger, 5 and 10 years in men and 5 and 8 years in women, respectively.

To take into account the potential confounding by smoking, we repeated our analyses by including only never smokers at baseline. Healthy LE and chronic disease-free LE were on average one year longer across all BMI categories in all cohorts and thus the gradient in decreasing healthy LE and chronic disease-free LE with increasing BMI remained similar as in the total population (Supplementary eTables 1 and 2).

Finally, we tested the sex × BMI interaction in multinomial logistic models. In most cases, this did not significantly improve model fit. However, the interaction was significant for self-rated health in GAZEL where the increased risk of remaining in poor health in the class II obesity was more marked in women than in men (*P*=0.013).

## Discussion

This study examined how BMI status was associated with healthy and chronic disease-free LE in men and women in four different cohorts from the England, Finland, France and Sweden. Our findings showed clear differences between BMI categories indicating that normal weight and overweight people could expect to live longest and normal weight people would spend about 81% and overweight 76% of those years in good health. Although the LE among class I and class II obese was on average only one year less than that among the normal weight, class II obese people could expect to live 6–7 fewer years in good health and 7–9 fewer years without chronic diseases compared to those with normal weight.

To our knowledge, this is the first prospective study to provide health expectancy estimates for both self-rated health and chronic diseases across BMI categories by applying multistate models to longitudinal data to obtain transition probabilities between health states. By performing detailed inspection of the WHO's BMI classification and splitting obesity category (BMI⩾30 kg m^−2^) into two obesity subcategories (class I and II obese) we demonstrated important variation in LE and health expectancies. Although there was only 0.5 year difference in LE from ages 50 to 75 years between class I and class II obese, the difference in healthy and chronic disease-free LE was about 4 years. Similar loss of health expectancy at higher obesity classes was also observed in a recent cross-sectional analysis.^16^ The heterogeneity within the group of obese people was also observed in the European Community Household Panel study in which disability-free LE across BMI categories was evaluated.^17^

In contrast to some previous studies which have reported longer LE among overweight than normal weight adults,^14, 16^ we did not observe marked LE difference between these groups. In a recent meta-analysis overweight was associated with lower all-cause mortality among subjects aged ⩾65 years old,^5^ but in the current study the participants were on average younger than 60 years at baseline. This difference in results may be explained by a greater potential for confounding by illness in older age groups. With advancing age the prevalence of many diseases increase which may lead to weight loss, thus making normal weight appear disadvantageous in terms of LE at older ages. Despite similar LE between normal weight and overweight groups, our analysis showed that normal weight participants had more healthy years and years without chronic diseases than overweight participants even after taking into account smoking status. Similar findings related to beneficial role of normal weight have been reported in terms of LE without cardiovascular disease^14, 22^ and disability.^17^

The findings of the study are highly relevant because together with an increase in obesity prevalence, the average number of years of life have continuously increased with no apparent plateau in Western countries.^39, 40^ The main question is how healthily the gained years of life will be spent. We showed consistent results across four different European countries suggesting that the obesity epidemic is likely to considerably decrease the proportion of life that is spent in good health. This has important implications for individuals and societies.^41^

The current study has a number of strengths. The major one is that our data are based on large prospective cohorts from four European countries, with multiple measurements of self-rated health and chronic diseases over time, long follow-up and high-quality harmonized data. In addition, we used microsimulation to estimate healthy LE and chronic disease-free LE which provides internally consistent results for each cohort.

The study also has a number of limitations that needs to be considered when interpreting the findings. First, although all cohorts showed consistent patterns of decreasing years of good health from normal weight to class II obese, the absolute difference in the years varied slightly across cohorts and by the health indicator. Despite careful harmonization there was some heterogeneity in the definitions of health and chronic diseases between cohort studies and the cohorts were also different in terms of representativeness and age.^42^ Indeed, ELSA is the only study including a national representative sample of older individuals, whereas FPS, GAZEL and SLOSH are occupational cohorts including individuals initially healthy enough to be in the labor force. However, the fact that the main findings were similar across different source populations suggests that the associations observed are robust and not restricted to some population segments. Second, BMI was based on objectively measured body weight and height only in ELSA. All other cohorts relied on self-reported information, suggesting that the data could be subject to potential measurement errors which hampers comparisons between cohorts.

Previous studies have shown that overweight and obese persons tend to underreport their weight which may lead to underestimation of BMI and potential misclassification of some individuals into incorrect BMI categories.^43, 44^ Despite to this potential misclassification towards lower BMI categories in other cohorts than ELSA, the data from WHO's database suggests that adult obesity prevalence is highest in England (24% in men and 26% in women), followed by Finland (21 and 19%), Sweden (13 and 12%) and France (9 and 8%),^45^ which corresponds with BMI distribution in our study cohorts. In addition, previous studies have shown that health risk estimates associated with BMI values are very similar, whether based on self-report or measured BMI values.^46^ Thus, it is unlikely that different ways of obtaining BMI data have markedly influenced on our main findings. Third, the number of underweight participants was too low to derive reliable estimates of its effect on healthy and chronic disease-free LE and thus this group was excluded from our analyses and should be examined in future studies with larger sample sizes. Finally, our life expectancy analyses were conditional on reaching age of 50 and truncated at age 75. Future studies are needed to investigate the association of BMI and healthy and chronic disease-free LE starting at younger ages and extending follow-up beyond the age of 75 years.

In conclusion, data from four European countries show that obese and severely obese people can expect to live much higher proportion of their life in sub-optimal health and with chronic diseases compared to normal weight people. Given the large impact of obesity on the burden of ill-health and the general increase in life expectancy, slowing down the obesity epidemic at the population level is essential to increase the time spent in good health.

## Figures and Tables

**Table 1:** Characteristics of the study cohorts at the time of first observation^a^

	*ELSA*		*FPS*		*GAZEL*		*SLOSH*	
	*Men*	*Women*	*Men*	*Women*	*Men*	*Women*	*Men*	*Women*
Sample size	4071	4733	8343	34 173	11 098	3833	3663	4326
								
Age (mean, s.d.)	61.5 (7.2)	61.4 (7.3)	53.6 (3.2)	53.2 (2.9)	52.0 (2.2)	51.4 (2.0)	58.0 (5.8)	57.3 (5.7)

*Socioeconomic position (%)*								
High grade	36.5	24.9	42	27	32.8	9.2	22.8	17.1
Middle grade	19.8	27.3	24.1	56.4	55.3	67.7	36.5	51.7
Low grade	43.8	47.8	33.9	16.6	11.8	23.1	40.7	31.1
Sub-optimal self-rated health (%)	25.2	23.8	37.3	34.1	19.2	23.5	23.6	20.3
Chronic disease (%)^b^	34.4	31.3	25.5	26	23.1	25.3	21.7	17.2

*Body mass index (%)*								
Underweight (<18.5 kg m^−2^)	0.3	0.9	0.2	0.8	0.3	2.9	0.2	0.9
Normal weight (18.5–24.9 kg m^−2^)	23.5	32	35.4	51.1	44.1	69.8	33.6	52.5
Overweight (25–29.9 kg m^−2^)	52.3	38.3	49	33.7	48.3	20.4	52	34.2
Obese class I (30–34.9 kg m^−2^)	19.2	19.3	12.4	11	6.5	5.5	12	9.7
Obese class II (⩾35 kg m^−2^)	4.8	9.6	3	3.4	0.7	1.6	2.2	2.7

*Smoking*								
Never smoking, %	27.4	43.5	50.7	67.7	35.2	67.9	52.7	50.4
Former smoking, %	53.5	36	27.7	17	35.3	11.8	32.1	31.3
Current smoking, %	19.1	20.5	21.6	15.3	29.6	20.3	15.3	18.3

a The first observation point refers to the date each participant was included in the dataset.

b Presence of chronic diseases includes illness reported at or before the first observation point.

**Table 2:** Partial life expectancy, healthy life expectancy and unhealthy life expectancy based on self-reported health between ages 50 and 75 years by body mass index in each study cohort

	*Life expectancy*	*95% CI*		*Healthy life expectancy*	*95% CI*		*Unhealthy life expectancy*	*95% CI*	
*Men*									
ELSA									
Normal weight	23.83	23.46	24.05	18.98	18.37	19.64	4.86	4.26	5.24
Overweight	24.01	23.83	24.25	18.68	18.3	19.22	5.32	4.92	5.7
Obese class I	23.45	23.21	23.82	16.37	15.8	17.12	7.08	6.39	7.67
Obese class II	23.17	22.36	23.48	13.07	11.54	13.93	10.1	9.07	11.34
FPS									
Normal weight	24.26	24.12	24.58	18.23	17.82	18.56	6.03	5.81	6.52
Overweight	24.26	24.03	24.47	15.21	14.73	15.55	9.05	8.71	9.51
Obese class I	23.53	23.22	24.04	11.43	11.05	12.54	12.1	11.29	12.55
Obese class II	22.76	21.8	23.51	7.97	6.57	8.79	14.79	13.7	16.16
GAZEL									
Normal weight	24.56	24.43	24.72	21.13	20.9	21.34	3.43	3.29	3.6
Overweight	24.57	24.45	24.71	20.43	20.23	20.62	4.14	3.97	4.32
Obese class I	23.69	23.22	24.16	18.16	17.61	18.84	5.53	5.09	5.99
Obese class II	23.35	21.65	24.54	14.65	12.93	16.63	8.7	6.94	9.81
SLOSH									
Normal weight	25.26	24.81	25.53	20.91	20.25	21.55	4.35	3.75	4.85
Overweight	25.23	24.94	25.49	19.21	18.6	19.87	6.03	5.45	6.47
Obese class I	25.7	25.29	26	15.96	14.79	17.07	9.74	8.66	10.98
Obese class II	24.22	21.99	26	13.72	10.99	16.32	10.5	7.89	12.96

*Women*									
ELSA									
Normal weight	24.59	24.43	24.8	20.08	19.72	20.62	4.51	4.12	4.81
Overweight	24.71	24.56	24.87	19.36	18.75	19.79	5.36	5	5.89
Obese class I	24.35	24.14	24.56	17.19	16.5	17.74	7.16	6.71	7.79
Obese class II	23.87	23.51	24.31	13.06	11.86	14.15	10.81	9.91	11.96
FPS									
Normal weight	25.05	24.95	25.14	18.41	18.28	18.69	6.63	6.38	6.78
Overweight	25.01	24.91	25.13	15.46	15.24	15.77	9.54	9.26	9.79
Obese class I	24.72	24.47	24.87	12.47	11.85	12.72	12.25	11.93	12.85
Obese class II	24.12	23.7	24.62	9.06	8.23	9.93	15.05	14.18	16
GAZEL									
Normal weight	25.07	24.85	25.16	20.54	20.26	20.81	4.53	4.24	4.71
Overweight	24.92	24.79	25.21	19.63	19.3	20.09	5.3	4.98	5.67
Obese class I	24.58	24.11	24.93	17.64	16.87	18.43	6.94	6.3	7.56
Obese class II	24.18	23.01	25.13	13.94	12.32	15.73	10.24	8.39	11.79
SLOSH									
Normal weight	25.45	25.21	25.68	21.67	21.13	22.12	3.78	3.43	4.35
Overweight	25.45	25.14	25.7	19.9	19.27	20.43	5.54	5.07	6.11
Obese class I	25.74	25.3	26	17.02	15.51	18.36	8.72	7.59	10.23
Obese class II	24.84	23.73	26	15.83	13.71	18.16	9	6.81	11.21

Abbreviation: CI, confidence interval.

**Table 3:** Partial life expectancy, chronic disease-free life expectancy and life expectancy with chronic diseases between ages 50 and 75 years by body mass index in each study cohort

	*Life expectancy*	*95% CI*		*Chronic disease-free life expectancy*	*95% CI*		*Life expectancy with chronic diseases*	*95% CI*	
*Men*									
ELSA									
Normal weight	23.62	23.24	23.89	14.2	12.93	15.54	9.42	8.08	10.63
Overweight	23.91	23.7	24.25	14.12	13.1	15.46	9.79	8.56	10.79
Obese class I	23.53	23.06	23.78	12.44	11.06	13.89	11.08	9.67	12.38
Obese class II	23	22.39	23.68	8.88	6.59	10.88	14.12	12.3	16.34
FPS									
Normal weight	24.16	23.98	24.44	14.44	13.97	14.96	9.72	9.23	10.28
Overweight	24.25	24	24.41	12.66	12.03	12.98	11.59	11.24	12.2
Obese class I	23.55	23.11	24.03	9.33	8.2	9.65	14.22	13.83	15.64
Obese class II	22.23	21.62	23.52	5.99	5.15	7.25	16.24	15.11	17.85
GAZEL									
Normal weight	24.53	24.4	24.7	15.52	15.15	15.83	9.01	8.73	9.41
Overweight	24.58	24.41	24.7	13.97	13.69	14.37	10.61	10.18	10.87
Obese class I	23.78	23.29	24.2	10.78	10.1	11.86	13	11.79	13.7
Obese class II	23.37	21.65	24.38	5.32	3.81	7.43	18.04	15.22	19.65
SLOSH									
Normal weight	25.19	24.74	25.53	16.35	15.26	17.57	8.85	7.56	9.95
Overweight	25.19	24.92	25.5	14.42	13.5	15.31	10.77	9.86	11.72
Obese class I	25.66	25.27	26	9.86	8.46	11.47	15.8	14.29	17.2
Obese class II	24.33	21.31	26	6.53	4.79	10.28	17.79	14.12	20.24

*Women*									
ELSA									
Normal weight	24.48	24.27	24.69	15.81	14.72	17.09	8.67	7.43	9.66
Overweight	24.62	24.47	24.83	15	13.88	16.65	9.63	8.09	10.82
Obese class I	24.38	24.09	24.58	13.85	12.18	15.45	10.53	8.98	12.1
Obese class II	23.95	23.49	24.32	9.5	7.84	11.74	14.45	12.06	15.96
FPS									
Normal weight	24.98	24.92	25.1	14.67	14.39	14.95	10.32	10.02	10.62
Overweight	24.94	24.89	25.12	12.73	12.39	13.04	12.2	11.97	12.62
Obese class I	24.54	24.43	24.86	10.02	9.56	10.48	14.52	14.14	15.15
Obese class II	24.17	23.62	24.58	7.29	6.69	8.29	16.88	15.78	17.53
GAZEL									
Normal weight	25.06	24.88	25.22	15.8	15.38	16.35	9.26	8.69	9.64
Overweight	25	24.8	25.19	13.96	13.1	14.73	11.04	10.37	11.88
Obese class I	24.33	24.02	24.9	11.16	10.13	12.51	13.18	12.02	14.26
Obese class II	24.35	22.86	24.99	8.37	5.52	10.65	15.98	13.15	18.84
SLOSH									
Normal weight	25.46	25.22	25.65	18.5	17.6	19.14	6.96	6.27	7.78
Overweight	25.42	25.18	25.69	15.45	14.35	16.37	9.97	9.02	11.05
Obese class I	25.82	25.48	26	11.39	9.87	12.93	14.43	12.74	15.99
Obese class II	24.73	23.28	26	10.27	7.34	13.28	14.45	11.75	17.72

Abbreviation: CI, confidence interval.

## References

[bib1] Ng M, Fleming T, Robinson M, Thomson B, Graetz N, Margono C et al. Global, regional, and national prevalence of overweight and obesity in children and adults during 1980-2013: a systematic analysis for the Global Burden of Disease Study 2013. Lancet 2014; 384: 766–781. 24880830 10.1016/S0140-6736(14)60460-8 PMC4624264

[bib2] National Institutes of HealthClinical Guidelines on the Identification, Evaluation, and Treatment of Overweight and Obesity in Adults: The Evidence Report. National Institutes of Health, NHLBI: Bethesda, MD, USA, 1998. 9813653

[bib3] Aune D, Sen A, Norat T, Janszky I, Romundstad P, Tonstad S et al. Body mass index, abdominal fatness, and heart failure incidence and mortality: a systematic review and dose-response meta-analysis of prospective studies. Circulation 2016; 133: 639–649. 26746176 10.1161/CIRCULATIONAHA.115.016801

[bib4] Renehan AG, Tyson M, Egger M, Heller RF, Zwahlen M. Body-mass index and incidence of cancer: a systematic review and meta-analysis of prospective observational studies. Lancet 2008; 371: 569–578. 18280327 10.1016/S0140-6736(08)60269-X

[bib5] Flegal KM, Kit BK, Orpana H, Graubard BI. Association of all-cause mortality with overweight and obesity using standard body mass index categories: A systematic review and meta-analysis. JAMA 2013; 309: 71–82. 23280227 10.1001/jama.2012.113905 PMC4855514

[bib6] Aune D, Sen A, Prasad M, Norat T, Janszky I, Tonstad S et al. BMI and all cause mortality: systematic review and non-linear dose-response meta-analysis of 230 cohort studies with 3.74 million deaths among 30.3 million participants. BMJ 2016; 353: i2156. 27146380 10.1136/bmj.i2156 PMC4856854

[bib7] Coggon D, Reading I, Croft P, McLaren M, Barrett D, Cooper C. Knee osteoarthritis and obesity. Int J Obes Relat Metab Disord 2001; 25: 622–627. 11360143 10.1038/sj.ijo.0801585

[bib8] Stenholm S, Rantanen T, Alanen E, Reunanen A, Sainio Pi, Koskinen S. Obesity history as a predictor of walking limitation at old age. Obesity 2007; 15: 929–938. 17426328 10.1038/oby.2007.583

[bib9] Alley DE, Chang VW. The changing relationship of obesity and disability, 1988–2004. JAMA 2007; 298: 2020–2027. 17986695 10.1001/jama.298.17.2020

[bib10] Larsson U, Karlsson J, Sullivan M. Impact of overweight and obesity on health-related quality of life—a Swedish population study. Int J Obes Relat Metab Disord 2002; 26: 417–424. 11896499 10.1038/sj.ijo.0801919

[bib11] Zaninotto P, Pierce M, Breeze E, de Oliveira C, Kumari M. BMI and waist circumference as predictors of well-being in older adults: findings from the English Longitudinal Study of Ageing. Obesity (Silver Spring) 2010; 18: 1981–1987. 20075853 10.1038/oby.2009.497

[bib12] Sanders BS. Measuring community health levels. Am J Public Health Nations Health 1964; 54: 1063–1070. 14157838 10.2105/ajph.54.7.1063 PMC1254928

[bib13] Wood R, Sutton M, Clark D, McKeon A, Bain M. Measuring inequalities in health: the case for healthy life expectancy. J Epidemiol Community Health 2006; 60: 1089–1092. 17108308 10.1136/jech.2005.044941 PMC2465513

[bib14] Nusselder WJ, Franco OH, Peeters A, Mackenbach JP. Living healthier for longer: comparative effects of three heart-healthy behaviors on life expectancy with and without cardiovascular disease. BMC Public Health 2009; 9: 487. 20034381 10.1186/1471-2458-9-487 PMC2813239

[bib15] van Baal PH, Hoogenveen RT, de Wit GA, Boshuizen HC. Estimating health-adjusted life expectancy conditional on risk factors: results for smoking and obesity. Popul Health Metr 2006; 4: 14. 17083719 10.1186/1478-7954-4-14 PMC1636666

[bib16] Steensma C, Loukine L, Orpana H, Lo E, Choi B, Waters C et al. Comparing life expectancy and health-adjusted life expectancy by body mass index category in adult Canadians: a descriptive study. Popul Health Metr 2013; 11: 21. 24252500 10.1186/1478-7954-11-21 PMC3842774

[bib17] Majer IM, Nusselder WJ, Mackenbach JP, Kunst AE. Life Expectancy and Life Expectancy With Disability of Normal Weight, Overweight, and Obese Smokers and Nonsmokers in Europe. Obesity 2011; 19: 1451–1459. 21415846 10.1038/oby.2011.46

[bib18] Walter S, Kunst A, Mackenbach J, Hofman A, Tiemeier H. Mortality and disability: the effect of overweight and obesity. Int J Obes 2009; 33: 1410–1418. 10.1038/ijo.2009.176 19786964

[bib19] Reuser M, Bonneux LG, Willekens FJ. Smoking kills, obesity disables: a multistate approach of the US Health and Retirement Survey. Obesity (Silver Spring) 2009; 17: 783–789. 19165165 10.1038/oby.2008.640

[bib20] Fransen HP, May AM, Beulens JW, Struijk EA, de Wit GA, Boer JM et al. Association between lifestyle factors and quality-adjusted life years in the EPIC-NL cohort. PLoS ONE 2014; 9: e111480. 25369457 10.1371/journal.pone.0111480 PMC4219750

[bib21] Stenholm S, Head J, Kivimäki M, Kawachi I, Aalto V, Zins M et al. Smoking, physical inactivity and obesity as predictors of healthy and disease-free life expectancy between ages 50 and 75: a multicohort study. Int J Epidemiol 2016; 45: 1260–1270. 27488415 10.1093/ije/dyw126 PMC6937009

[bib22] O'Doherty MG, Cairns K, O'Neill V, Lamrock F, Jorgensen T, Brenner H et al. Effect of major lifestyle risk factors, independent and jointly, on life expectancy with and without cardiovascular disease: results from the Consortium on Health and Ageing Network of Cohorts in Europe and the United States (CHANCES). Eur J Epidemiol 2016; 31: 455–468. 26781655 10.1007/s10654-015-0112-8 PMC4901087

[bib23] Sullivan D. A single index of mortality and morbidity. HSMHA Health Rep 1971; 86: 347–354. 5554262 PMC1937122

[bib24] Cai L. The cost of an additional disability-free life year for older Americans: 1992-2005. Health Services Res 2013; 48: 218–235. 10.1111/j.1475-6773.2012.01432.x PMC3589963 22670874

[bib25] Pongiglione B, De Stavola BL, Ploubidis GB. A systematic literature review of studies analyzing inequalities in health expectancy among the older population. PLoS One 2015; 10: 6. 10.1371/journal.pone.0130747 PMC4482630 26115099

[bib26] Collaboration PS. Body-mass index and cause-specific mortality in 900 000 adults: collaborative analyses of 57 prospective studies. Lancet 2009; 373: 1083–1096. 19299006 10.1016/S0140-6736(09)60318-4 PMC2662372

[bib27] Steptoe A, Breeze E, Banks J, Nazroo J. Cohort Profile: The English Longitudinal Study of Ageing. Int J Epidemiol 2013; 42: 1640–1648. 23143611 10.1093/ije/dys168 PMC3900867

[bib28] Goldberg M, Leclerc A, Zins M. Cohort profile update: The GAZEL Cohort Study. Int J Epidemiol 2015; 44: 77g. 25422284 10.1093/ije/dyu224

[bib29] Magnusson Hanson LL, Theorell T, Oxenstierna G, Hyde M, Westerlund H. Demand, control and social climate as predictors of emotional exhaustion symptoms in working Swedish men and women. Scand J Public Health 2008; 36: 737–743. 18684778 10.1177/1403494808090164

[bib30] World Health OrganizationObesity: preventing and managing the global epidemic Report of a WHO Consultation. WHO Technical Report Series: Geneva, Switzerland, 894 2000. Contract No.: Report. 11234459

[bib31] Niedhammer I, Chea M. Psychosocial factors at work and self reported health: comparative results of cross sectional and prospective analyses of the French GAZEL cohort. Occup Environ Med 2003; 60: 509–515. 12819285 10.1136/oem.60.7.509 PMC1740565

[bib32] Cai L, Hayward MD, Saito Y, Lubitz J, Hagedorn A, Crimmins E. Estimation of multi-state life table functions and their variability from complex survey data using the SPACE Program. Demogr Res 2010; 22: 129–158. 20463842 10.4054/DemRes.2010.22.6 PMC2867357

[bib33] Pirie K, Peto R, Reeves GK, Green J, Beral V. The 21st century hazards of smoking and benefits of stopping: a prospective study of one million women in the UK. Lancet 2013; 381: 133–141. 23107252 10.1016/S0140-6736(12)61720-6 PMC3547248

[bib34] Carter BD, Abnet CC, Feskanich D, Freedman ND, Hartge P, Lewis CE et al. Smoking and mortality—beyond established causes. N Engl J Med 2015; 372: 631–640. 25671255 10.1056/NEJMsa1407211

[bib35] Winslow UC, Rode L, Nordestgaard BG. High tobacco consumption lowers body weight: a Mendelian randomization study of the Copenhagen General Population Study. Int J Epidemiol 2015; 44: 540–550. 25777141 10.1093/ije/dyu276

[bib36] Cai LM, Hayward MD, Saito Y, Lubitz J, Hagedorn A, Crimmins E. Estimation of multi-state life table functions and their variability from complex survey data using the SPACE Program. Demogr Res 2010; 22: 129–157. 20463842 10.4054/DemRes.2010.22.6 PMC2867357

[bib37] Centers for Disease Control and Prevention. SPACE Program 2015 [cited 2016, accessed on 15 June 2016]. Available from http://www.cdc.gov/nchs/data_access/space.htm.

[bib38] Lièvre A, Brouard M, Heathcote C. The estimation of health expectancies from cross-longitudinal surveys. Math Popul Stud 2003; 10: 211–248.

[bib39] Christensen K, Doblhammer G, Rau R, Vaupel JW. Ageing populations: the challenges ahead. Lancet 2009; 374: 1196–1208. 19801098 10.1016/S0140-6736(09)61460-4 PMC2810516

[bib40] Vaupel JW. Biodemography of human ageing. Nature 2010; 464: 536–542. 20336136 10.1038/nature08984 PMC4010874

[bib41] Beltran-Sanchez H, Soneji S, Crimmins EM. Past, Present, and Future of Healthy Life Expectancy. Cold Spring Harb Perspect Med 2015; 5. 10.1101/cshperspect.a025957 PMC4632858 26525456

[bib42] Pongiglione B, De Stavola BL, Ploubidis GB. A systematic literature review of studies analyzing inequalities in health expectancy among the older population. PLoS One 2015; 10: e0130747. 26115099 10.1371/journal.pone.0130747 PMC4482630

[bib43] Connor Gorber S, Tremblay M, Moher D, Gorber B. A comparison of direct vs. self-report measures for assessing height, weight and body mass index: a systematic review. Obes Rev 2007; 8: 307–326. 17578381 10.1111/j.1467-789X.2007.00347.x

[bib44] Nyholm M, Gullberg B, Merlo J, Lundqvist-Persson C, Rastam L, Lindblad U. The validity of obesity based on self-reported weight and height: Implications for population studies. Obesity (Silver Spring) 2007; 15: 197–208. 17228048 10.1038/oby.2007.536

[bib45] WHO Global Infobase [Internet]. 2016 [cited 12 May 2016]. Available from https://apps.who.int/infobase/Index.aspx.

[bib46] Stommel M, Schoenborn CA. Accuracy and usefulness of BMI measures based on self-reported weight and height: findings from the NHANES & NHIS 2001-2006. BMC Public Health 2009; 9: 421. 19922675 10.1186/1471-2458-9-421 PMC2784464

