# Supplementary material for: Body mass index as a predictor of healthy and disease-free life expectancy between ages 50 and 75: a multicohort study
Source: Int J Obes (Lond). 2017 Feb 21;41(5):769–75. doi: 10.1038/ijo.2017.29 (PMC5418561; doi:10.1038/ijo.2017.29)
Supplement: Supplementary Information [file ijo201729x1.docx]

**Supplementary material**

**eTable 1. Partial life expectancy, healthy life expectancy and unhealthy life expectancy based on self-reported health between the ages of 50 and 75 by body mass index among never smokers in each study cohort.**

|  | Life expectancy | 95% CI | |  | Healthy life expectancy | 95% CI | |  | Unhealthy life expectancy | 95% CI | |  | %* | 95% CI | |
| --- | --- | --- | --- | --- | --- | --- | --- | --- | --- | --- | --- | --- | --- | --- | --- |
| **MEN** |  |  |  |  |  |  |  |  |  |  |  |  |  |  |  |
| ELSA |  |  |  |  |  |  |  |  |  |  |  |  |  |  |  |
| Normal weight | 24.71 | 24.43 | 25.27 |  | 21.74 | 21.2 | 22.4 |  | 2.97 | 2.54 | 3.52 |  | 88 | 85.9 | 89.7 |
| Overweight | 24.79 | 24.61 | 25.09 |  | 20.89 | 20.28 | 21.57 |  | 3.9 | 3.26 | 4.33 |  | 84.3 | 82.4 | 86.9 |
| Obese class I | 24.31 | 23.96 | 24.94 |  | 18.24 | 17.05 | 19.41 |  | 6.07 | 5.1 | 7.04 |  | 75 | 70.8 | 79 |
| Obese class II | 23.48 | 22.73 | 24.23 |  | 14.6 | 13.33 | 16.49 |  | 8.88 | 6.87 | 10.06 |  | 62.2 | 56.7 | 70 |
|  |  |  |  |  |  |  |  |  |  |  |  |  |  |  |  |
| FPS |  |  |  |  |  |  |  |  |  |  |  |  |  |  |  |
| Normal weight | 24.91 | 24.69 | 25.15 |  | 19.69 | 19.28 | 20.24 |  | 5.22 | 4.94 | 5.6 |  | 79.1 | 77.6 | 80.4 |
| Overweight | 24.79 | 24.46 | 24.99 |  | 16.81 | 16.37 | 17.25 |  | 7.98 | 7.52 | 8.38 |  | 67.8 | 66.2 | 69.5 |
| Obese class I | 24.09 | 23.53 | 24.53 |  | 13.11 | 12.31 | 13.7 |  | 10.98 | 10.08 | 11.77 |  | 54.4 | 51.5 | 57.5 |
| Obese class II | 23.13 | 22.34 | 24.48 |  | 9.23 | 7.58 | 10.79 |  | 13.9 | 12.17 | 15.8 |  | 39.9 | 32.9 | 46.3 |
|  |  |  |  |  |  |  |  |  |  |  |  |  |  |  |  |
| GAZEL |  |  |  |  |  |  |  |  |  |  |  |  |  |  |  |
| Normal weight | 25.06 | 24.85 | 25.24 |  | 21.95 | 21.79 | 22.14 |  | 3.11 | 2.92 | 3.23 |  | 87.6 | 87.2 | 88.4 |
| Overweight | 24.87 | 24.72 | 25.03 |  | 21.08 | 20.84 | 21.25 |  | 3.79 | 3.64 | 3.96 |  | 84.8 | 84 | 85.4 |
| Obese class I | 24.24 | 23.64 | 24.61 |  | 19.36 | 18.75 | 20.01 |  | 4.87 | 4.44 | 5.18 |  | 79.9 | 78.4 | 81.8 |
| Obese class II | 24.19 | 23.07 | 26 |  | 16.63 | 14.95 | 18.4 |  | 7.56 | 5.78 | 9.39 |  | 68.8 | 62.9 | 75.6 |
|  |  |  |  |  |  |  |  |  |  |  |  |  |  |  |  |
| SLOSH |  |  |  |  |  |  |  |  |  |  |  |  |  |  |  |
| Normal weight | 25.50 | 25.04 | 25.92 |  | 21.7 | 21.16 | 22.37 |  | 3.8 | 3.36 | 4.31 |  | 85.1 | 83.2 | 87 |
| Overweight | 25.42 | 24.97 | 25.84 |  | 20.2 | 19.61 | 20.84 |  | 5.22 | 4.56 | 5.81 |  | 79.5 | 77.3 | 82.1 |
| Obese class I | 25.74 | 25.05 | 26 |  | 17.64 | 16.52 | 18.82 |  | 8.09 | 7.02 | 9.15 |  | 68.6 | 64.6 | 72.8 |
| Obese class II | 21.41 | 15.4 | 25.16 |  | 13.58 | 9.88 | 17.94 |  | 7.83 | 5.46 | 14.6 |  | 63.4 | 42 | 74.5 |
|  |  |  |  |  |  |  |  |  |  |  |  |  |  |  |  |
| **WOMEN** |  |  |  |  |  |  |  |  |  |  |  |  |  |  |  |
| ELSA |  |  |  |  |  |  |  |  |  |  |  |  |  |  |  |
| Normal weight | 25.32 | 25.18 | 25.58 |  | 21.89 | 21.15 | 22.51 |  | 3.43 | 2.85 | 4.14 |  | 86.5 | 83.6 | 88.8 |
| Overweight | 25.37 | 25.2 | 25.49 |  | 21 | 20.3 | 21.67 |  | 4.38 | 3.72 | 4.9 |  | 82.8 | 80.6 | 85.3 |
| Obese class I | 25.01 | 24.88 | 25.3 |  | 17.98 | 16.81 | 19.32 |  | 7.04 | 5.9 | 8.15 |  | 71.9 | 67.3 | 76.6 |
| Obese class II | 24.53 | 24.32 | 25.02 |  | 14.8 | 13.05 | 16.78 |  | 9.73 | 8.15 | 11.39 |  | 60.3 | 53.4 | 67.3 |
|  |  |  |  |  |  |  |  |  |  |  |  |  |  |  |  |
| FPS |  |  |  |  |  |  |  |  |  |  |  |  |  |  |  |
| Normal weight | 25.31 | 25.22 | 25.45 |  | 19.19 | 18.88 | 19.34 |  | 6.12 | 6.03 | 6.35 |  | 75.8 | 74.8 | 76.2 |
| Overweight | 25.18 | 25.08 | 25.3 |  | 16.1 | 15.88 | 16.38 |  | 9.08 | 8.84 | 9.35 |  | 63.9 | 62.9 | 64.9 |
| Obese class I | 24.68 | 24.51 | 24.97 |  | 12.62 | 12.1 | 13.01 |  | 12.06 | 11.69 | 12.76 |  | 51.1 | 48.7 | 52.7 |
| Obese class II | 24.27 | 23.69 | 25 |  | 9.41 | 8.69 | 10.5 |  | 14.86 | 13.93 | 15.79 |  | 38.8 | 35.8 | 42.6 |
|  |  |  |  |  |  |  |  |  |  |  |  |  |  |  |  |
| GAZEL |  |  |  |  |  |  |  |  |  |  |  |  |  |  |  |
| Normal weight | 25.07 | 25.01 | 25.28 |  | 20.73 | 20.62 | 21.06 |  | 4.34 | 4.16 | 4.52 |  | 82.7 | 82.1 | 83.5 |
| Overweight | 24.94 | 24.83 | 25.37 |  | 19.74 | 19.45 | 20.14 |  | 5.21 | 5.05 | 5.61 |  | 79.1 | 77.7 | 79.9 |
| Obese class I | 24.48 | 23.96 | 24.87 |  | 18.05 | 17.46 | 18.78 |  | 6.43 | 5.84 | 6.81 |  | 73.7 | 71.9 | 76.3 |
| Obese class II | 24.31 | 23.78 | 26 |  | 15.15 | 13.56 | 16.96 |  | 9.16 | 7.44 | 11.67 |  | 62.3 | 54.4 | 69.5 |
|  |  |  |  |  |  |  |  |  |  |  |  |  |  |  |  |
| SLOSH |  |  |  |  |  |  |  |  |  |  |  |  |  |  |  |
| Normal weight | 25.72 | 25.6 | 25.97 |  | 22.31 | 21.92 | 22.81 |  | 3.41 | 3 | 3.9 |  | 86.7 | 84.9 | 88.4 |
| Overweight | 25.71 | 25.11 | 25.95 |  | 20.77 | 19.55 | 21.42 |  | 4.94 | 4.28 | 6.04 |  | 80.8 | 76.4 | 83.4 |
| Obese class I | 25.89 | 25.59 | 26 |  | 18.64 | 16.97 | 20.16 |  | 7.25 | 5.84 | 8.76 |  | 72 | 66 | 77.6 |
| Obese class II | 23.46 | 20.42 | 25.46 |  | 16.35 | 13.26 | 19.08 |  | 7.11 | 4.37 | 11.5 |  | 69.7 | 54.5 | 80.9 |
|  |  |  |  |  |  |  |  |  |  |  |  |  |  |  |  |

*Notes*: * Proportion of life spent in good health between the ages of 50 and 75.

**eTable 2. Partial life expectancy, chronic disease-free life expectancy and life expectancy without chronic diseases between the ages of 50 and 75 by body mass index among never smokers in each study cohort.**

|  | Life expectancy | 95% CI | |  | Chronic disease-free life expectancy | 95% CI | |  | Life expectancy with chronic diseases | 95% CI | |  | %* | 95% CI | |
| --- | --- | --- | --- | --- | --- | --- | --- | --- | --- | --- | --- | --- | --- | --- | --- |
| **MEN** |  |  |  |  |  |  |  |  |  |  |  |  |  |  |  |
| ELSA |  |  |  |  |  |  |  |  |  |  |  |  |  |  |  |
| Normal weight | 24.61 | 24.32 | 24.88 |  | 15.65 | 12.26 | 17.89 |  | 8.95 | 6.85 | 12.62 |  | 63.6 | 49.3 | 72.3 |
| Overweight | 24.69 | 24.43 | 24.9 |  | 15.13 | 11.41 | 15.96 |  | 9.56 | 8.69 | 13.37 |  | 61.3 | 46 | 64.7 |
| Obese class I | 24.38 | 23.9 | 24.57 |  | 14.3 | 10.43 | 15.06 |  | 10.08 | 9.34 | 13.8 |  | 58.6 | 43.2 | 61.8 |
| Obese class II | 23.49 | 22.7 | 24.2 |  | 10 | 5.64 | 14.6 |  | 13.49 | 8.7 | 18.25 |  | 42.6 | 23.7 | 62.9 |
|  |  |  |  |  |  |  |  |  |  |  |  |  |  |  |  |
| FPS |  |  |  |  |  |  |  |  |  |  |  |  |  |  |  |
| Normal weight | 24.77 | 24.67 | 25.11 |  | 14.92 | 14.58 | 15.63 |  | 9.85 | 9.21 | 10.34 |  | 60.2 | 58.6 | 62.7 |
| Overweight | 24.67 | 24.49 | 24.98 |  | 13.28 | 12.44 | 13.83 |  | 11.39 | 11.06 | 12.21 |  | 53.8 | 50.5 | 55.5 |
| Obese class I | 23.82 | 23.52 | 24.55 |  | 9.6 | 8.73 | 10.41 |  | 14.22 | 13.49 | 15.6 |  | 40.3 | 36.1 | 43.3 |
| Obese class II | 23.89 | 22.42 | 24.76 |  | 7.49 | 5.7 | 9.43 |  | 16.4 | 14.22 | 18.07 |  | 31.4 | 24.1 | 39.9 |
|  |  |  |  |  |  |  |  |  |  |  |  |  |  |  |  |
| GAZEL |  |  |  |  |  |  |  |  |  |  |  |  |  |  |  |
| Normal weight | 25.04 | 24.88 | 25.15 |  | 17.01 | 16.49 | 17.44 |  | 8.03 | 7.59 | 8.52 |  | 67.9 | 65.9 | 69.7 |
| Overweight | 24.82 | 24.71 | 25.12 |  | 15.67 | 15.14 | 15.99 |  | 9.15 | 8.88 | 9.62 |  | 63.1 | 61.2 | 64.3 |
| Obese class I | 24.22 | 23.76 | 25.04 |  | 12.51 | 11.13 | 13.4 |  | 11.71 | 10.7 | 12.98 |  | 51.6 | 46.8 | 55.5 |
| Obese class II | 24.14 | 23.15 | 25.14 |  | 8.93 | 5.68 | 12.44 |  | 15.22 | 11.64 | 18.97 |  | 37 | 23 | 51 |
|  |  |  |  |  |  |  |  |  |  |  |  |  |  |  |  |
| SLOSH |  |  |  |  |  |  |  |  |  |  |  |  |  |  |  |
| Normal weight | 25.54 | 25.26 | 25.8 |  | 16.89 | 15.88 | 18.63 |  | 8.65 | 7.15 | 9.67 |  | 66.1 | 62.2 | 72.2 |
| Overweight | 25.45 | 25.17 | 25.79 |  | 15.61 | 14.53 | 16.45 |  | 9.84 | 8.86 | 11.1 |  | 61.3 | 56.7 | 65 |
| Obese class I | 25.81 | 25.41 | 26 |  | 10.21 | 8.87 | 11.61 |  | 15.6 | 14.23 | 17.13 |  | 39.6 | 34.1 | 44.9 |
| Obese class II | 21.36 | 13.34 | 24.79 |  | 6.81 | 3.43 | 11.15 |  | 14.55 | 6.32 | 19.24 |  | 31.9 | 21.3 | 58.1 |
|  |  |  |  |  |  |  |  |  |  |  |  |  |  |  |  |
| **WOMEN** |  |  |  |  |  |  |  |  |  |  |  |  |  |  |  |
| ELSA |  |  |  |  |  |  |  |  |  |  |  |  |  |  |  |
| Normal weight | 25.25 | 25.09 | 25.46 |  | 16.87 | 13.37 | 18.59 |  | 8.38 | 6.61 | 11.71 |  | 66.8 | 53.3 | 73.8 |
| Overweight | 25.28 | 25.08 | 25.4 |  | 15.21 | 13.05 | 17.25 |  | 10.07 | 8.11 | 12.28 |  | 60.2 | 51.6 | 68 |
| Obese class I | 25.06 | 24.83 | 25.29 |  | 15.06 | 12.31 | 16.67 |  | 10 | 8.53 | 12.64 |  | 60.1 | 49.3 | 66.2 |
| Obese class II | 24.64 | 24.19 | 25.05 |  | 12.16 | 9.68 | 16.21 |  | 12.49 | 8.82 | 14.86 |  | 49.3 | 39.6 | 64.8 |
|  |  |  |  |  |  |  |  |  |  |  |  |  |  |  |  |
| FPS |  |  |  |  |  |  |  |  |  |  |  |  |  |  |  |
| Normal weight | 25.3 | 25.17 | 25.35 |  | 15.17 | 14.96 | 15.61 |  | 10.13 | 9.68 | 10.35 |  | 60 | 59.1 | 61.7 |
| Overweight | 25.17 | 25.1 | 25.28 |  | 13.22 | 12.94 | 13.73 |  | 11.95 | 11.48 | 12.3 |  | 52.5 | 51.3 | 54.5 |
| Obese class I | 24.69 | 24.29 | 24.95 |  | 10.14 | 9.6 | 10.8 |  | 14.55 | 13.95 | 15.22 |  | 41.1 | 38.9 | 43.6 |
| Obese class II | 24.43 | 23.69 | 24.81 |  | 7.59 | 7.07 | 8.6 |  | 16.84 | 15.72 | 17.36 |  | 31.1 | 29 | 35 |
|  |  |  |  |  |  |  |  |  |  |  |  |  |  |  |  |
| GAZEL |  |  |  |  |  |  |  |  |  |  |  |  |  |  |  |
| Normal weight | 25.1 | 24.96 | 25.22 |  | 17.11 | 16.64 | 17.66 |  | 7.99 | 7.35 | 8.4 |  | 68.2 | 66.5 | 70.6 |
| Overweight | 24.96 | 24.69 | 25.21 |  | 14.9 | 14.14 | 15.48 |  | 10.06 | 9.5 | 10.84 |  | 59.7 | 56.7 | 61.8 |
| Obese class I | 24.4 | 23.59 | 24.91 |  | 12.23 | 10.92 | 13.22 |  | 12.16 | 11.09 | 13.7 |  | 50.1 | 44.4 | 54 |
| Obese class II | 24.43 | 23.36 | 25.03 |  | 10.45 | 8.47 | 13.45 |  | 13.98 | 10.05 | 15.69 |  | 42.8 | 35.5 | 57.2 |
|  |  |  |  |  |  |  |  |  |  |  |  |  |  |  |  |
| SLOSH |  |  |  |  |  |  |  |  |  |  |  |  |  |  |  |
| Normal weight | 25.77 | 25.52 | 25.97 |  | 18.47 | 17.28 | 19.07 |  | 7.3 | 6.78 | 8.39 |  | 71.7 | 67.3 | 73.8 |
| Overweight | 25.75 | 25.54 | 25.99 |  | 16.5 | 15.58 | 17.02 |  | 9.25 | 8.62 | 10.41 |  | 64.1 | 60 | 66.6 |
| Obese class I | 25.89 | 25.78 | 26 |  | 11.95 | 9.49 | 14.04 |  | 13.95 | 11.95 | 16.49 |  | 46.1 | 36.5 | 54 |
| Obese class II | 23.98 | 22.44 | 25.94 |  | 10.06 | 7.67 | 14.31 |  | 13.92 | 9.33 | 16.77 |  | 41.9 | 33.3 | 60.7 |
|  |  |  |  |  |  |  |  |  |  |  |  |  |  |  |  |

*Notes*: * Proportion of life spent without chronic diseases between the ages of 50 and 75.
